# Meta-Analysis of Data from Four Clinical Trials in the Ivory Coast Assessing the Efficacy of Two Artemisinin-Based Combination Therapies (Artesunate-Amodiaquine and Artemether-Lumefantrine) between 2009 and 2016

**DOI:** 10.3390/tropicalmed9010010

**Published:** 2023-12-29

**Authors:** Akoua Valérie Bédia-Tanoh, Kondo Fulgence Kassi, Offianan André Touré, Serge Brice Assi, Akpa Paterne Gnagne, Koffi Daho Adoubryn, Emmanuel Bissagnene, Abibatou Konaté, Jean Sebastien Miezan, Kpongbo Etienne Angora, Henriette Vanga-Bosson, Pulchérie Christiane Kiki-Barro, Vincent Djohan, William Yavo, Eby Ignace Hervé Menan

**Affiliations:** 1 Parasitology and Mycology Departement, Training and Research Unit of Pharmaceutical and Biological Sciences, University Félix Houphouët-Boigny, Abidjan P.O. Box V34, Côte d’Ivoire; 2 Malaria Research and Control Center/National Institute of Public Health, Abidjan P.O. Box V47, Côte d’Ivoire; gap75m@yahoo.fr; 3 Diagnostic and Research Center on AIDS and Others Infectious Diseases (CeDReS), University Hospital Center, Treichville, Abidjan P.O. Box V13, Côte d’Ivoire; 4 Malariology Department Institut Pasteur of Ivory Coast, Abidjan P.O. Box 490, Côte d’Ivoire; 5 Scientific Advisory Board of National Malaria Control Program, Abidjan P.O. Box V4, Côte d’Ivoire; 6 Pierre Richet Institute (IPR) Bouake/National Institute of Public Health, Bouaké P.O. Box 1500, Côte d’Ivoire; 7 Parasitology and Mycology Department, Training and Research Unit of Medical Sciences, University Alassane Ouattara, Bouaké P.O. Box 1801, Côte d’Ivoire

**Keywords:** malaria, artesunate-amodiaquine, artemether-lumefantrine, efficacy

## Abstract

The combinations of artemether-lumefantrine (AL) and artesunate-amodiaquine (ASAQ) are used as first-line treatments for uncomplicated malaria in the Ivory Coast. Different studies document the efficacy of two artemisinin-based combination therapies (ACTs) (AL and ASAQ) in the Ivory Coast. However, there is no meta-analysis examining the data set of these studies. The purpose of this work was to determine the prevalence of malaria treatment failure cases in randomized control trials with two artemisinin-based combination therapies (AL versus ASAQ) in the Ivory Coast between 2009 to 2016. This study is a meta-analysis of data from the results of four previous multicenter, open-label, randomized clinical trial studies evaluating the clinical and parasitological efficacy of artemether-lumefantrine and artesunate-amodiaquine conducted between 2009 and 2016 following World Health Organization (WHO) protocol at sentinel sites in the Ivory Coast. These drug efficacy data collected between 2009 and 2016 were analyzed. During these studies, to distinguish between recrudescence and new infection, molecular genotyping of genes encoding merozoite surface protein 1 and 2 was carried out using nested polymerase chain reaction (PCR). A total of 1575 patients enrolled in the four studies, including 768 in the AL arm and 762 in the ASAQ arm, which were fully followed either for 28 days or 42 days according to WHO protocol. The adequate clinical and parasitological response (ACPR) was higher than 95% in the two groups (intention to treat (ITT): AL = 96.59% and ASAQ = 96.81; Per Protocol (PP): AL = 99.48% and ASAQ = 99.61%) after PCR correction at day 28. Aggregate data analysis (2009–2016) showed that at day 28, the proportions of patients with recurrent infection was higher in the AL group (ITT: 3.79%, PP: 3.9%) than in the ASAQ group (ITT: 2.17%, PP: 2.23%). After PCR correction, most treatment failures were classified as new infections (AL group (ITT: 0.13%, PP: 0.13%); ASAQ group (ITT: 0.39%, PP: 0.39%). The recrudescent infections rate was high, at 0.39% compared to 0.13% for ASAQ and AL, respectively, for both ITT and PP, no significant difference. However, the Kaplan–Meier curve of cumulative treatment success showed a significant difference between the two groups after PCR from 2012–2013 (*p* = 0.032). Overall, ASAQ and AL have been shown to be effective drugs for the treatment of uncomplicated *P. falciparum* malaria in the study areas, 14 years after deployment of these drugs.

## 1. Introduction

Malaria remains a significant public health concern worldwide, particularly in sub-Saharan Africa. It is a leading cause of morbidity and mortality in Africa. In 2021, the World Health Organization (WHO) estimated that there were approximately 247 million malaria cases, 95% of which occurred in the African region, with 619,000 deaths [1]. Children under five years old and pregnant women are the most impacted by this infection [2]. In the Ivory Coast, the malaria transmission is perennial with seasonal peaks during the rainy seasons (the long rainy season (mid-March to mid-July), and the short rainy season (September to mid-November)). *Plasmodium falciparum* is the most common species found (95–99%) in the Ivory Coast [3,4]. Malaria is the leading cause of outpatient visits (43%), as well the leading cause of morbidity (40%) and mortality (10%) in the general population [4,5]. In Africa, *Plasmodium falciparum* resistance to the widely used drugs in monotherapies in uncomplicated malaria is at a very high level, and this has hampered malaria control efforts in certain countries. As a consequence, the use of combination regimens against malaria has been widely advocated by the World Health Organization [6]. Now, combination regimens are implemented in the majority of endemic African countries [4,7,8,9,10], including the Ivory Coast [3,11]. They are the mainstay of treatment for malaria globally. In combination with other strategies, the advances in the control of malaria are partly related to use of artemisinin-based combination therapies (ACTs) [12].

Since 2005, artemisinin-based combination therapies (ACTs) have been used in the Ivory Coast for the treatment of uncomplicated malaria [5,12]. These were artesunate-amodiaquine in first-line treatment and artemether-lumefantrine combination in second-line treatment. Now, artemether–lumefantrine (AL), artesunate–amodiaquine (ASAQ), dihydroartemisinin-piperaquine (DHAP), and artesunate-pyronaridine (AP) are recommended in first intention therapy in this country [13]. In the WHO’s African region, these have efficacy rates against *Plasmodium falciparum* infections of 98%, 98.4%, and 99.4%, respectively, which have not declined over time [14]. Multiple clinical trials have shown both AL and ASAQ to offer excellent efficacy for the treatment of malaria in Africa

However, there is a threat of artemisinin resistance in malaria endemic regions as previously described in South East Asia [7,15]. Artemisinin resistance, manifested by delayed parasite clearance after treatment, is growing, and because of the possibility of extension to other endemic areas, it is a serious threat [16,17]. Thus, the WHO recommends that the efficacy of the first- and second-line antimalarial drugs should be regularly assessed every two years in the malaria control and elimination strategy [18]. This assessment will enable early detection and prevention of the spread of resistant parasite populations [18].

Since implementing the national protocol using ACTs, multiple clinical trials have shown both AL and ASAQ taken individually offer the best efficacy malaria treatment in the Ivory Coast [3,4,19,20]. Nevertheless, at the country level, some delays in the parasite clearance of ACTs have been reported [3,11,19]. However, there is no meta-analysis examining the dataset of these studies. The purpose of this work was to determine prevalence of malaria treatment failure cases in randomized control trials with two artemisinin-based combination therapies (AL versus ASAQ) in the Ivory Coast between 2009 and 2016.

## 2. Materials and Methods

### 2.1. Study Area

Therapeutic efficacy studies (TESs) of artemether–lumefantrine and artesunate–amodiaquine were conducted in the Ivory Coast between 2009 and 2016. The study areas were Dabakala, Ayamé, Abengourou, San-Pedro, Yamoussoukro, Abobo, Man, Korogho, and Abidjan. These study areas varied across the four studies.

- The first study was conducted from 2008 to 2009 in the health district of Dabakala, located in the central-eastern part of the Ivory Coast and Ayamé.
- The second study was conducted in 2012 in three sites: the health districts of Abengourou, located in the southeastern forest region, San Pedro in the southwest coastal and forest region, and Yamoussoukro in the country’s central Lake District.
- The third study occurred in the cities of Man, Korhogo, Abengourou, San-Pedro, Yamoussoukro, and Abidjan, in 2013.
- The last study was conducted in Abidjan, Man, Abengourou, San-Pedro, Yamoussoukro, and Korhogo from 2015 to 2016.

**Figure 1:**
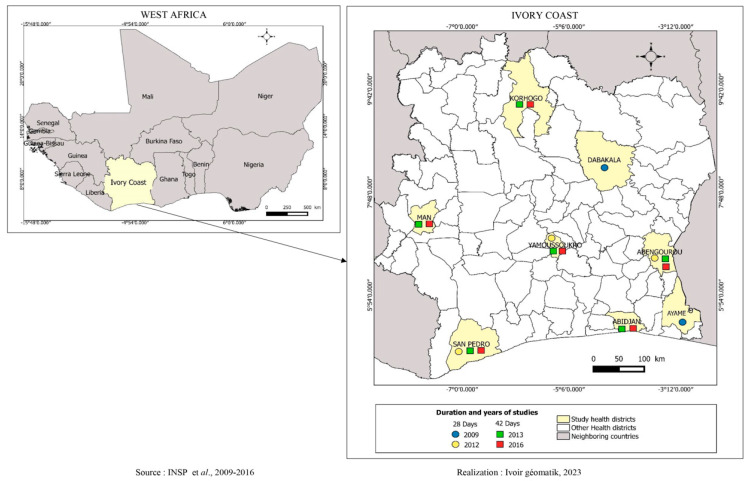
Map displaying AL and ASAQ efficacy study sites by the study period and the number of days of patient follow-up.

Figure 1 shows the study sites.

### 2.2. Study Design

This study is a meta-analysis of data from the results of four previous multicenter, open-label, and randomized clinical trial studies evaluating the clinical and parasitological efficacy of artemether-lumefantrine and artesunate-amodiaquine conducted between 2009 and 2016 following WHO protocol at sentinel sites in the Ivory Coast, by the National Malaria Control Program (NMCP) [3,4]. This study was conducted for a more cogent analysis of the data by increasing the number of case studies in order to increase the statistical power to find significant results and obtain a closer estimation to the effect of the practical use of artemether-lumefantrine and artesunate-amodiaquine.

### 2.3. Study Participants and Inclusion Criteria

The study population consisted of outpatients who were presented to health facilities with uncomplicated malaria symptoms. Patients were referred to the study team for recruitment. If patients met the inclusion criteria below, they were included in the study [3,4,8].

- Inclusion criteria;
- Patients aged over six months;
- Without distinction by sex (male or female patient);
- Presenting fever with an axillary temperature of ≥37.5 °C or a history of fever within 24 h;
- With acute uncomplicated *P. falciparum* malaria (2000–200,000 parasites/μL), confirmed under the microscope (Giemsa-stained thick and thin films);
- Having the ability to ingest tablets orally;
- Patients having provided their consent to participate in the study or parents or guardians consented to children participating in the study.
- Exclusion criteria;
- Severe malnutrition;
- Pregnant women and nursing mothers;
- Signs of altered general condition or signs of severe malaria;
- Fever due to a disease other than malaria;
- History of a hypersensitive reaction to the combination of artemether–lumefantrine and/or artesunate–amodiaquine or to one of the constituents;
- Taking an antimalarial drug (or a drug with known antiplasmodial activity) in the week preceding inclusion.

For cases of therapeutic failure or protocol violation, the patients were treated according to the NMCP guidelines by the local team.

### 2.4. Randomization, Treatment, and Follow-Up

The patients were randomly assigned to receive either the ASAQ or AL drugs administered under the research teams’ supervision, according to their body weight for three consecutive days. The follow-up period was different for the different studies. Thus, the recruited subjects were followed up at either 28 days (D28) or 42 days (D42) following the WHO standardized 28 day or 42 day protocol [8]. Three drops of blood were collected on filter paper (Whatman international Ltd., Maidstone, UK) on day 0 and in the case of fever or parasitemia recurrence the parasite DNA was extracted [20] and genotyped by nested PCR [21] to compare the polymorphism in the merozoite surface protein genes 1 and 2 to distinguish the new infection from recrudescence.

### 2.5. Data Collection

A new database (Excel) was developed from the digital data obtained by various investigators from the following research institutions where the studies were conducted:

The Unit of Paludology of the Institut Pasteur of the Ivory Coast in 2009, 2013, and 2016;

The Department of Parasitology-Mycology of the Pedagogic Training Unit of Pharmaceutical Sciences and Biology in 2012 and 2016;

The Department of Parasitology–Mycology of the Pedagogic Training Unit of Medical Sciences and the Institut Pierre Richet of Bouaké in 2016.

The data collected during each survey were compiled.

### 2.6. Outcome Assessment

Efficacy outcomes were based on the WHO definitions [8]: 1. early treatment failure (ETF), 2. late treatment failure (LTF), 3. late parasitological failure (LPF), and 4. adequate clinical and parasitological response (ACPR).

### 2.7. Statistical Analysis

The information collected from the various studies’ digital databases was compiled to create a single database with Microsoft Excel 2013 software. All data were recorded using IBM Epi-info version 7. The Kaplan-Meier curve from survival analysis was displayed using Graphpad Prism. Comparing different parameters in both arms was performed using the chi-square test or Fisher’s exact test at the 5% significance level. The intention-to-treat analysis included all randomized participants who took at least one full dose without a major protocol violation. Cases of protocol violation, lost to follow-up, or withdrawal from the study were considered therapeutic failures. The per-protocol data included all participants who received the three ASAQ or six AL doses without protocol violation up to day 28 or 42.

The data obtained were classified according to the duration of the investigation protocol of 28 days or 42 days. The classification allowed us to identify three periods of study. The first one from 2009 to 2012 had an investigation protocol of 28 days, the second period from 2013 to 2016 had an investigation protocol of 42 days, and the third one from 2009 to 2016 was the compilation of the periods of 2009–2012 (28 days) and 2013–2016 (42 days) following the protocol of investigation of 28 days (aggregated data analysis).

The recurrence is defined as a new clinical manifestation of the infection after the initial removal of parasites in the peripheral blood. In case of reinfection (verified by PCR), parasitological recurrence is not considered as a treatment failure of the malaria drug received.

Parasite clearance time: time elapsed between the first administration and the first total and continued disappearance of parasite asexual forms, persisting for at least another 24 h. Thermal or fever clearance time: time elapsed between the first dose and the first lowering of the temperature below 37.5 °C for at least another 24 h.

## 3. Results

### 3.1. Baseline Characteristics

The clinical and parasitological characteristics of the patients are summarized in Table 1. In total, 1575 patients meeting the inclusion criteria were included for the period of 2009–2016, 784 in the ASAQ arm and 791 in the AL arm (Table 1). The baseline characteristics (age, sex, weight, axillary or rectal temperature, and parasitemia) in the intention-to-treat (ITT) cohort of the two groups were similar (Table 1).

### 3.2. Period of 2009–2012

Overall, 542 patients were randomized and enrolled into the two treatment groups. A total of 542,530 outcomes were available in the PP analysis. The adequate clinical parasitological response (ACPR) was good at day 28 in the two groups after PCR correction (ITT: ASAQ = 97.06% vs. AL = 97.78%; PP: ASAQ = 99.62% vs. AL = 100%) (Table 2).

The ITT therapeutic failure rate before PCR correction in the deux AL and ASAQ arms was, 4.09% and 1.83% at day 28, respectively, with no statistically significant difference observed.

The rate of therapeutic failure after PCR correction was identical in the two arms at day 28 (0.37%). The majority of failures observed before PCR correction were reinfestations. Most failure cases observed before the PCR correction were reinfection (Table 2).

The rate of treatment failure (FT) in PP before PCR correction was higher in the AL arm (4.15%) than in the ASAQ arm (1.51%), with no statistically significant difference. All the positive cases observed were reinfestations after PCR correction in the ASAQ arm. In the AL arm, only 0.38% were late parasitological failure (LPF).

### 3.3. Period of 2013–2016

Overall, 521 and 512 patients were randomized and enrolled into the ASAQ and AL groups, respectively. The ACRP at day 42 after PCR correction in the ITT analysis was 96.68% and 95.97% for the ASAQ and AL, respectively. In the PP analysis, this rate was 99.6% and 100% for the ASAQ and AL, respectively (Table 3).

The ITT therapeutic failure rate (TFR), before PCR correction in the AL and ASAQ arms was 5.18% and 3.71 at day 28, respectively. However, no statistically significant difference was observed.

Two cases of recrudescence (EPT and ETP) were noted in ITT, after PCR correction in the ASAQ arm. In the AL arm, all cases of failure were reinfestations after PCR correction. The therapeutic failure rate in PP before PCR correction in the AL and ASAQ arms, respectively, was 3.80% and 2.41% at day 28. At day 42, there were increased failure rates in both the AL (5.40%) and ASAQ arms and in the ASAQ arm (4.02%). However, no statistically significant difference was observed.

In per-protocol (PP) analysis, two cases of recrudescence were observed in the ASAQ arm after PCR correction. In the AL arm, on the other hand, all cases of failure observed before PCR correction turned out to be reinfestations after PCR correction (Table 3).

**Figure 2:**
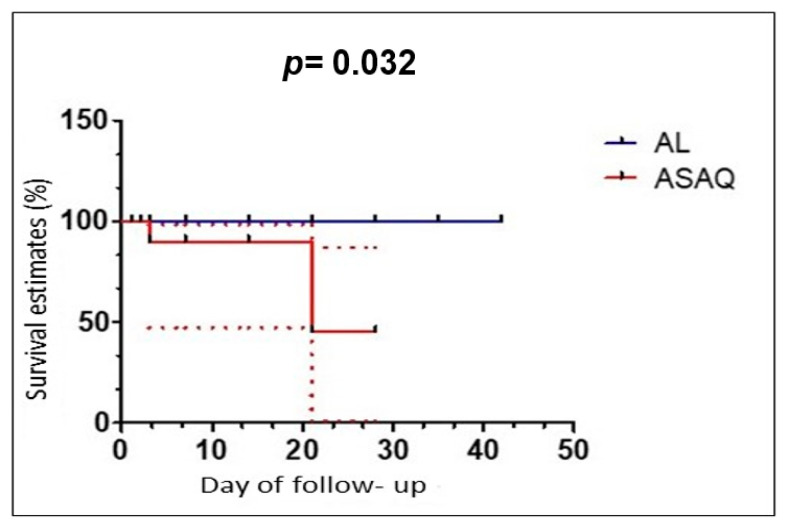
PCR-corrected survival curve from 2013–2016.

Figure 2 shows the Kaplan-Meier curve of the cumulative treatment success over the 42-day follow-up period, after PCR correction. The follow-up rate of the patients treated with AL was 100% compared to 46.1% for the group treated with ASAQ at 42 days. There was a statistically significant difference between the follow-up rate and the drugs used (*p* = 0.032) (Figure 2).

### 3.4. Period of 2009–2016

The ITT therapeutic failure rate before PCR correction in the two arms AL and ASAQ was 3.79% and 2.17%, respectively, at day 28. After PCR correction, there was a reduction in the failure rate in both arms (AL: 0.13%; ASAQ: 0.39%). However, the failure rate observed in the ASAQ arm was higher than in the AL arm, with no statistically significant difference. The therapeutic failure rate in PP before PCR correction in the two arms AL and ASAQ, was 3.9% and 2.23%, respectively, at day 28. However, no statistically significant difference was observed. The rate of therapeutic failure in PP, after PCR correction in the AL and ASAQ arms, decreased in both the AL and ASAQ arms by 0.13% and 0.39%, respectively, at day 28 but was higher in the AL arm. No statistically significant difference was observed.

**Figure 3:**
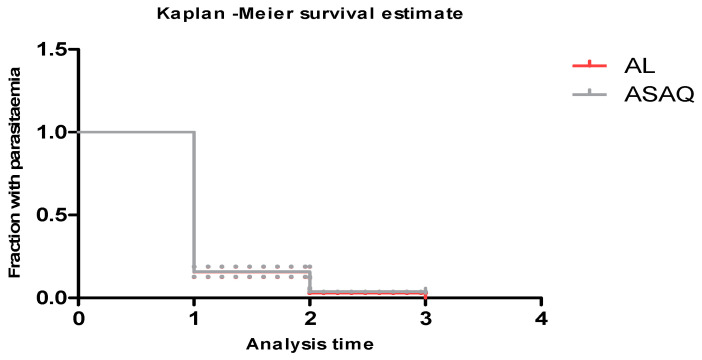
Clearance time of parasitemia by day of study patients.

**Figure 4:**
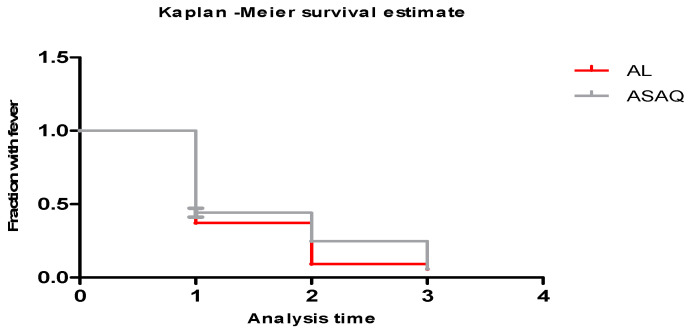
Clearance time of fever by day of study patients.

The clearance times and fever clearance were comparable between the two study arms (Figure 3 and Figure 4). Both treatments produced a rapid clearance of parasites (Figure 3) on day two at 99% for the ASAQ and AL groups. On day three, the parasite clearance rate was 100% in both groups (Figure 3). The fever clearance was observed on day two after the first dose treatment (Figure 4).

The adequate clinical and parasitological response (ACPR) was higher than 95% in the two groups (ITT: AL = 96.59% and ASAQ = 96.81; PP: AL = 99.48% and ASAQ = 99.61%) after PCR correction at day 28 (Table 4). The results showed that the failure rate observed in the ASAQ group was superior to that of the AL group (ITT and PP: AL = 0.13%; ASAQ = 0.39%). No statistically significant difference was observed (Table 4).

**Figure 5:**
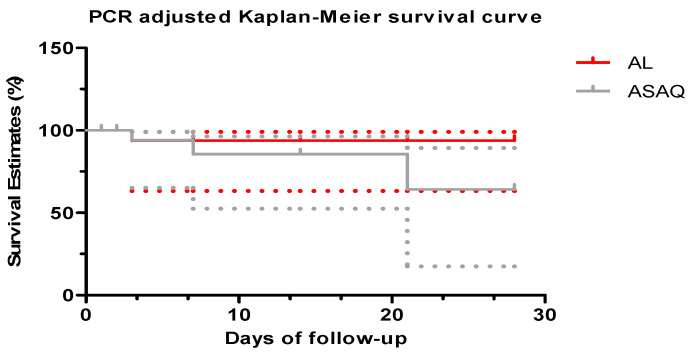
Kaplan-Meier curves PCR adjusted (2009–2016).

Figure 5 shows the Kaplan-Meier curve of the cumulative treatment success over the 28-day follow-up period after PCR correction. The follow-up rate of the patients treated with AL was 94% compared to 64% for the group treated by ASAQ at 28 days. There was no statistically significant difference between the follow-up rate and the medical devices used (*p* = 0.3) (Figure 5).

## 4. Discussion

This study was conducted to provide evidence of the clinical efficacy of ASAQ and AL, antimalarial drugs used as the first-line treatment for malaria, since combination treatment was adopted and implemented as the antimalarial drug policy in the Ivory Coast in 2005 (12). Both forms of ACTs have been frequently used in health system services. Updated international guidelines advocate the need for regular monitoring of the ACTs to detect early signs of declining efficacy, which may have implications for policymakers. Furthermore, this study aimed to determine the prevalence of malaria treatment failure cases of AL and ASAQ and to verify the observations of some prescribers who reported that AL had more cases of failure than ASAQ.

A strength of this study is mainly the sample size (1575 patients).

Children aged 0–5 are the most vulnerable to malaria and, therefore, the most affected by it. In this study, the 0–5 age group was the most represented. The sex ratio obtained reflected that malaria affects both men and women [19,20,21]. Fever was the primary clinical sign found because one of the inclusion criteria was the presence of a temperature of ≥37.5 °C. Furthermore, fever is the primary clinical sign of malaria [19,22].

The results regarding the rapid clearance of the fever and parasites were similar for both ACTs for a three days treatment. There was no clinically significant difference in efficacy between the AL and ASAQ combinations. Similar results in parasite clearance and fever have been reported in Nigeria [6]. The fever clearance and parasitemia clearance already allow us to assess efficacy of the two associations. So, the ASAQ and AL associations present good antiparasitic activity in our country and in West Africa [3,4,23]. Both drug regimens tested in this study showed adequate clinical and parasitological efficacy with a cure rate of 95%. Previous studies conducted in the Ivory Coast [3,4,19] and other malaria-endemic regions of Africa [6,23,24] were in agreement with our results. They have shown an equally high cure rate. However, studies conducted in East Africa have shown a higher efficacy of AL than ASAQ [25,26]. Treatment combination therapies have significantly improved the treatment and control of malaria [2].

Almost all sub-Saharan African countries recommend either AL or ASAQ for the treatment of uncomplicated malaria [2]; hence, the emergence of artemisinin resistance in some countries is a matter of great concern [27,28]. Therefore, it is essential to monitor the effectiveness of the ACTs [2]. Most of these recurrent parasitemia samples are reclassified as reinfections based on PCR genotyping, especially with the AL. Furthermore, Kaplan–Meier curve analysis showed a significant difference between the two groups after PCR, indicating a lower recrudescence level in the AL group from 2012 to 2013. However, after aggregating the 2009–2016 data, there was no significant difference between the data.

The low failure rate observed in our study indicates the importance of the administrative supervision of the ACT. The observations conducted by clinicians can be explained by the longer half-life of amodiaquine (8 days) compared to lumefantrine (4–6 days) and poor patient compliance [11]. Indeed, patients who stay in the same environment during their treatment with ASAQ or AL can be exposed to new infested bites that can lead to relapses. The number of the times the pills are taken daily, twice a day (morning and afternoon) for AL with the first two doses taken at 8 h intervals makes compliance difficult, which is not the case with ASAQ (one dose per day) [11,26]. Inadequate dosing due to poor compliance could reduce the duration of the post-therapeutic prophylactic effect of these combinations, especially AL.

The PCR-corrected cure rates indicated that the actual efficacy was comparable between the two treatments. Both treatments were well above the 95% threshold recommended by the WHO for current treatments [8]. The excellent efficacies observed with ASAQ and AL at the current doses seem to demonstrate good activity against the asexual forms of the parasite. This study provided scientific evidence that can help supplement the existing data regarding the effectiveness of ACTs in the Ivory Coast. Furthermore, the results of this study confirm the policy decision to use ASAQ and AL in the Ivory Coast [3,5,11]. However, the presence of a number of therapeutic failures shows that these drugs, especially AL, have come under drug pressure. It would therefore be interesting to integrate or introduce other ACTs such as DHAP, whose efficacy has been proven by several studies, into the guidelines for the therapeutic management of malaria [29].

Since 2017, DHAP has been used as a first-line treatment for uncomplicated malaria. Today, four molecules are used in the treatment of uncomplicated malaria in Ivory Coast: artemether-lumefantrine (AL), artesunate-amodiaquine (ASAQ), dihydroartemisinin-piperaquine, and artesunate-pyronaridine (AP) are used as first-line treatments [12].

These observations have been different in other countries. For example, clinical trials have shown a lower risk of recurrent parasitemia after treatment with AL compared to ASAQ [26,30,31]. Similarly, most studies conducted elsewhere in Africa have shown similar results for AL and ASAQ [14,32,33,34], although other studies conducted in West Africa have shown that ASAQ was more effective than AL [3].

This difference can be due to the lower resistance levels to amodiaquine in West Africa compared to other regions. In Uganda, Yeka et al. observed fewer recurrences after ASAQ treatment compared to AL [23].

Although AL and ASAQ have successfully treated uncomplicated malaria with few post-treatment upsurges, changes in the two ACTs’ relative performance must cause the NMCP to be more vigilant, especially considering the very high incidence of malaria in the Ivory Coast. Widespread use of the two combinations or the introduction into the treatment regimen of other ACTs known to be effective in treating malaria may reduce the selection of resistant strains and maintain the effectiveness of these two regimes.

A strength of this study is mainly the sample size (1575 patients). However, it had limitations. It is a retrospective study, which resulted in the loss of some data. The number of days of follow-up differed from one study to another (28 days or 42 days); so, for the aggregated analysis, we have only taken into account data collected from day 0 to day 28. Despite these limitations, important information was obtained on the efficacity of AL and ASAQ. This information could help the NMCP in the formulation of appropriate therapeutic and control strategies in the Ivory Coast.

## 5. Conclusions

At the end of this work, we observed that the adequate clinical and parasitological response (ACPR) was higher than 95% in the two groups, ASAQ and AL, after PCR correction at day 28. ASAQ and AL have been shown to be safe and effective drugs for treating uncomplicated *Plasmodium falciparum* malaria in the study areas 14 years after the deployment of these drugs. Nevertheless, the efficacy of these two different forms of ACTs must be carefully monitored periodically, as important treatment failures can occur due to resistance and subtherapeutic levels caused by inadequate absorption, poor adherence to the drug, or poor compliance.

## Figures and Tables

**Table 1:** Baseline characteristics of study patients by treatment arm.

Characteristics	AL (n = 791)	ASAQ (n = 784)	*p*-Value
Sex (Sex-ratio)	(0.9)	(1)	1
M	374	392	
F	417	392	
Age, year mean (±SD)	9 (9.9)	9.2 (11.4)	1
[0–5]	427	401	
[6–15]	232	270	
>15	122	113	
Temperature °C mean (±SD)	38.4 (1)	38.5 (1)	0.9
Parasite density, parasites/μL, mean (±SD)	40,354.3 (52,446.9)	41,562.5 (58,417)	0.5

**Table 2:** Intention-to-treat and per-protocol analysis efficacy of ASAQ and AL to 28 days (2009–2012).

Treatment Outcome	AL (n_1_ = 270)	ASAQ (n_2_ = 272)	Odd Ratio (CI 95%)	*p*-Value
Lost to follow up	5 (2.57%)	7 (1.85%)	0.72 (0.20 2.52)	0.7897
Intention To Treat (ITT) analysis				
PCR unadjusted				
ETF	4 (1.50%)	2 (0.73%)	0.5 (0.06–3.17)	0.6857
LTF	6 (2.22%)	3 (1.10%)	0.5 (0.10–2.25)	0.5042
LPF	1 (0.37%)	0 (0%)	0 (0–17.29)	0.4990
ACPR	254 (94.07%)	260 (95.59%)	1.02 (0.79–1.30)	0.8457
PCR adjusted				
ETF	0 (0%)	1 (0.37%)	-	1
LTF	0 (0%)	0 (0%)	-	-
LPF	1 (0.37%)	0 (0%)	0 (0–17.29)	0.4990
ACPR	264 (97.78%)	264 (97.06%)	0.99 (0.78–1.27)	0.9993
Per-Protocol (PP) analysis				
PCR unadjusted				
ETF	4(1.51%)	1(0.38%)	0.25 (0.01–2.38)	0.3726
LTF	6 (2.26%)	3 (1.13%)	0.5 (0.10–2.27)	0.5041
LPF	1 (0.38%)	0 (0%)	0 (0–17.42)	1
ACPR	254 (95.85%)	261 (98.49%)	1.03 (0.80–1.32)	0.8746
PCR adjusted				
ETF	0 (0%)	0 (0%)	-	-
LTF	0 (0%)	0 (0%)	-	-
LPF	0 (0%)	1 (0.38%)	0 (0–17.42)	1
ACPR	265 (100%)	264 (99.62%)	1 (0.78–1.29)	0.9755

**Table 3:** Intention-to-treat and per-protocol analysis efficacy of ASAQ and AL to day 42 (2013–2016).

Treatment Outcome	AL (n_1_ = 521)	ASAQ (n_2_ = 512)	Odd Ratio (CI 95%)	*p*-Value
Lost to follow up	21 (4.03%)	15 (2.92%)	1.38 (0.67–2.84)	0.4459
Intention to Treat (ITT) analysis				
PCR unadjusted				
ETF	20 (3.84%)	13 (2.54%)	0.66 (0.31–1.41)	0.3302
LTF	7 (1.34%)	6 (1.17%)	0.87 (0.26–2.90)	0.9723
LPF	0 (0%)	1 (0.20%)		0.4961
ACPR	473 (90.79%)	477 (93.16%)	1.03 (0.86–1.23)	0.8083
PCR adjusted				
ETF	0 (0%)	0 (0%)		
LTF	0 (0%)	1 (0.20%)		0.4961
LPF	0 (0%)	1 (0.20%)		0.4961
ACPR	500 (95.97%)	495 (96.68%)	1.01 (0.84–1.20)	0.9692
Per-Protocol (PP) analysis				
PCR unadjusted				
ETF	20 (4%)	13 (2.61%)	0.64 (0.30–1.39)	0.3149
LTF	7 (1.40%)	6 (1.21%)	0.93 (0.29–2.87)	0.9885
LPF	0 (0%)	1 (0.20%)		0.4989
ACPR	473 (94.6%)	477 (95.98%)	1.01 (0.85–1.22)	0.9093
PCR unadjusted				
ETF	0 (0%)	1 (0.20%)		0.4989
LTF	0 (0%)	1 (0.20%)		0.4989
LPF	0 (0%)	0 (0%)		
ACPR	500 (100%)	495 (99.60%)	1 (0.83–1.19)	1

**Table 4:** Intention-to-treat and per-protocol analysis efficacy of AL and ASAQ to day 28 (2009–2016).

Treatment Outcome	AL (n_1_ = 791)	ASAQ (n_2_ = 784)	Odd Ratio (CI 95%)	*p*-Value
Lost to follow up	23 (2.91%)	22 (2.80%)	1.04 (0.55–1.95)	0.9731
	Intention To Treat (ITT) analysis			
	PCR unadjusted			
ETF	17 (2.15%)	9 (1.15%)	0.53 (0.22–1.27)	0.1815
LTF	12 (1.51%)	7 (0.89%)	0.59 (0.21–1.61)	0.3733
LPF	1 (0.13%)	1 (0.13%)	1.01 (0–36.88)	1
ACPR	738 (93.30%)	745 (95.03%)	1.02 (0.88–1.18)	0.8281
	PCR adjusted			
Lost to follow up	26 (3.28%)	22 (2.80%)	1.17 (0.64–2.16)	0.6951
ETF	0 (0%)	1 (0.13%)		0.4980
LTF	0 (0%)	1 (0.13%)		0.4980
LPF	1 (0.13%)	1 (0.13%)	1.01 (0–36.88)	1
ACPR	764 (96.59%)	759 (96.81%)	1 (0.87–1.16)	0.9971
	Per-Protocol (PP) analysis			
	PCR unadjusted			
ETF	17 (2.21%)	9 (1.18%)	0.53 (0.22–1.27)	0.808
LTF	12 (1.56%)	7 (0.92%)	0.59 (0.21–1.61)	0.3722
LPF	1 (0.13%)	1 (0.13%)	1.01 (0–36.85)	1
ACPR	738 (96.1%)	745 (97.77%)	1.02 (0.88–1.18)	0.8409
	PCR adjusted			
ETF	0 (0%)	1 (0.13%)		0.4983
LTF	0 (0%)	1 (0.13%)		0.4983
LPF	1 (0.13%)	1 (0.13%)	1.01 (0–36.85)	1
ACPR	764 (99.48%)	759 (99.61%)	0.98 (0.76–1.25)	0.8985

## Data Availability

Data are contained within the article.
